# A rare solitary fibrous tumor in the ischiorectal fossa: a case report

**DOI:** 10.1186/s40792-018-0533-1

**Published:** 2018-10-03

**Authors:** Kazuhiko Morikawa, Shinsuke Takenaga, Koichi Masuda, Asami Kano, Takao Igarashi, Hiroya Ojiri, Kaoru Ueda, Mamoru Ishiyama, Nei Fukasawa

**Affiliations:** 1 Department of Radiology, The Jikei University Katsushika Medical Center, 6-41-2, Aoto, Katsushika-ku, Tokyo, 125-8506 Japan; 2 Department of Radiology, The Jikei University School of Medicine, 3-25-8, Nishi- Shimbashi, Minato-ku, Tokyo, 105-8461 Japan; 3 Department of Gastroenterology and Hepatology, The Jikei University Katsushika Medical Center, 6-41-2, Aoto, Katsushika-ku, Tokyo, 125-8506 Japan; 4 Department of Surgery, The Jikei University Katsushika Medical Center, 6-41-2, Aoto, Katsushika-ku, Tokyo, 125-8506 Japan; 5 Department of Pathology, The Jikei University Katsushika Medical Center, 6-41-2, Aoto, Katsushika-ku, Tokyo, 125-8506 Japan

**Keywords:** Solitary fibrous tumor, Mesenchymal tumor, Hypervascular tumor, Ischiorectal fossa, Pararectal tumor, Biopsy, Surgical excision

## Abstract

**Background:**

A solitary fibrous tumor (SFT) is a rare mesenchymal tumor that occurs mostly in pleural sites, and an SFT occurring in the ischiorectal fossa is extremely rare. Because of the rarity, there are few reports detailing an SFT in the ischiorectal fossa.

**Case presentation:**

A pararectal tumor was incidentally found in a 42-year-old man during a routine medical examination. The patient had no symptoms and no previous medical history. In the physical examination, a smooth-margined and hard elastic mass was felt, and in a digital rectal examination, the rectal mucosa appeared normal. A computed tomography (CT) scan showed a 5-cm, well-defined, solid mass in the left ischiorectal fossa. Contrast-enhanced CT in the early phase showed intense heterogeneous enhancement that persisted during the delayed phase. T2-weighted images of magnetic resonance imaging yielded heterogeneous intermediate and low signal intensity. Intense arterial enhancement suggested a hypervascular nature, and persistent delayed enhancement and low signal bands on T2-weighted images suggested a fibrous component of the mass. An SFT was suspected. Most SFTs are benign but have malignant potential. Our patient did not hope for surgery if the tumor was benign; therefore, an ultrasound-guided transperineal core needle biopsy was performed to decide on a treatment strategy. Microscopic examination showed tumor cells appearing as spindle and fibroblast-like cells within a collagenous stroma. Immunohistochemistry identified CD34 and vimentin, supporting the diagnosis of an SFT. The patient consented to excision of the mass. He was placed in a prone jackknife position, and the tumor was removed transperineally using a posterior approach (modified Kraske procedure). The levator ani muscle, external sphincter muscles, and rectum were not involved and separated from the tumor. The tumor was successfully resected en bloc with no complications. Five uneventful days post surgery, the patient was discharged. There was no local recurrence during the year following surgery.

**Conclusion:**

Imaging findings reflect the tissue characterization such as hypervascularity and fibrous nature of SFTs. We have presented a rare case of an SFT in the ischiorectal fossa with useful imaging findings for diagnosis, treatment strategy, and successful surgical removal using a posterior approach.

## Background

A solitary fibrous tumor (SFT) is a rare type of mesenchymal tumor that was initially reported to occur in the pleura. SFTs are now known to occur throughout the body [1]. However, SFTs occurring in the ischiorectal fossa are extremely rare. Here, we present a rare case of an SFT in the ischiorectal fossa including its imaging findings useful for diagnosis, strategy of treatment, and operative method.

## Case presentation

A 42-year-old man presented to our outpatient clinic because of the presence of a pararectal tumor that was found incidentally during a routine medical examination. The patient had no urinary or gastrointestinal symptoms and no previous medical history. In the physical examination, a smooth-margined, hard elastic mass was felt, and in a digital rectal examination, the rectal mucosa appeared to be normal.

**Fig. 1:**
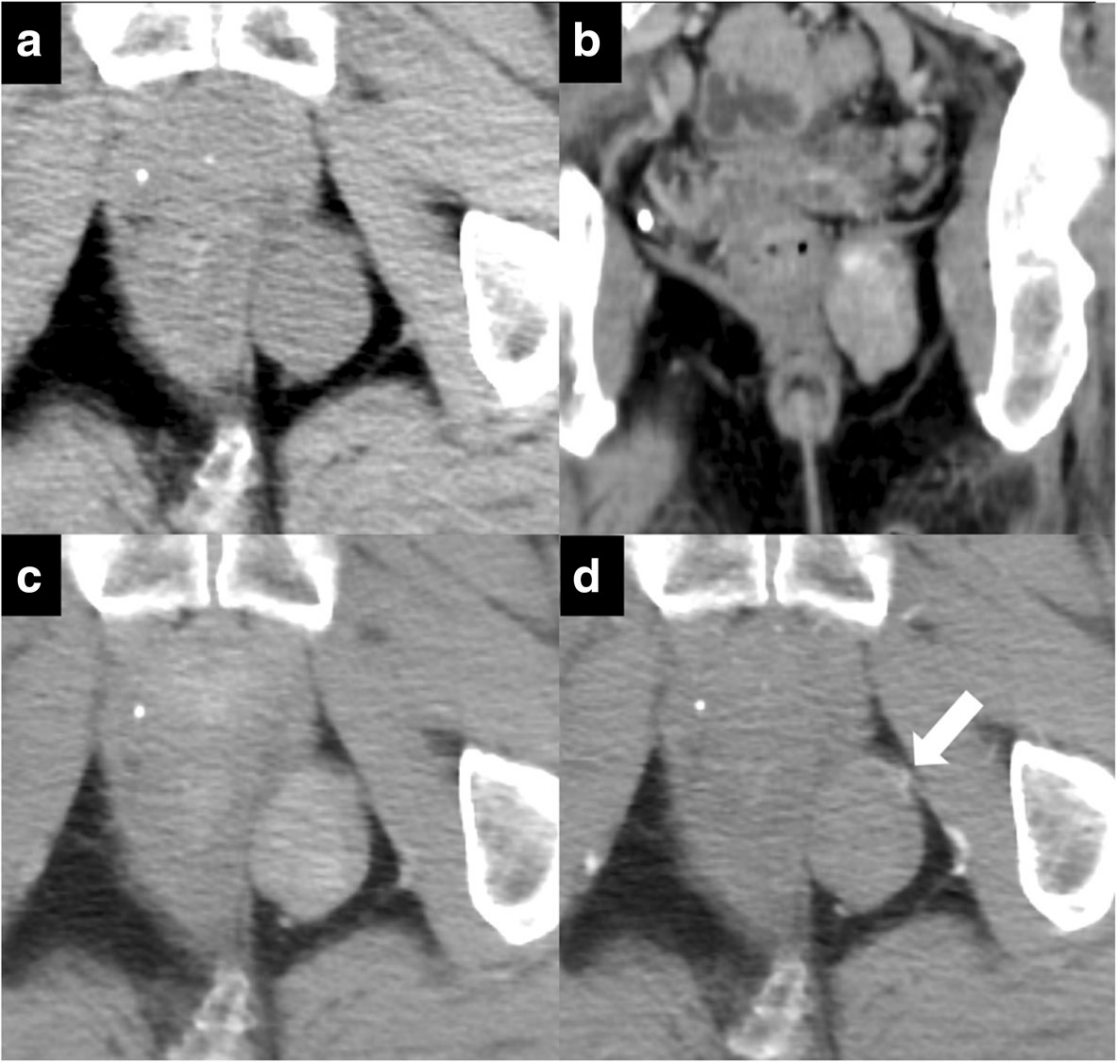
Axial and reconstruction of coronal contrast-enhanced CT. **a** A 5-cm, well-defined solid mass in the left ischiorectal fossa abutting the left anal wall and extending into the inferior perineum. **b** Coronal image: contrast-enhanced CT showing intense heterogeneous enhancement. **c** Axial image in the delayed phase. Delayed persistence of the mass is revealed. **d** Axial image in the early phase. A feeding vessel is seen near the mass (white arrow)

The computed tomography (CT) scan showed a 5-cm, well-defined, solid mass in the left ischiorectal fossa abutting the left anal wall and extending into the inferior perineum (Fig. 1a). Contrast-enhanced CT showed intense heterogeneous enhancement that persisted during the delayed phase (Fig. 1b, c), and a feeding vessel was visible around the mass (Fig. 1d). Intense arterial enhancement suggested a hypervascular nature and persistent delayed enhancement suggested a fibrous nature of the mass. The differential diagnosis was an SFT, gastrointestinal stromal tumor (GIST), aggressive angiomyxoma, leiomyoma, neurogenic tumor, or soft tissue sarcoma. There was no evidence of distant metastasis in the chest or abdomen.

**Fig. 2:**
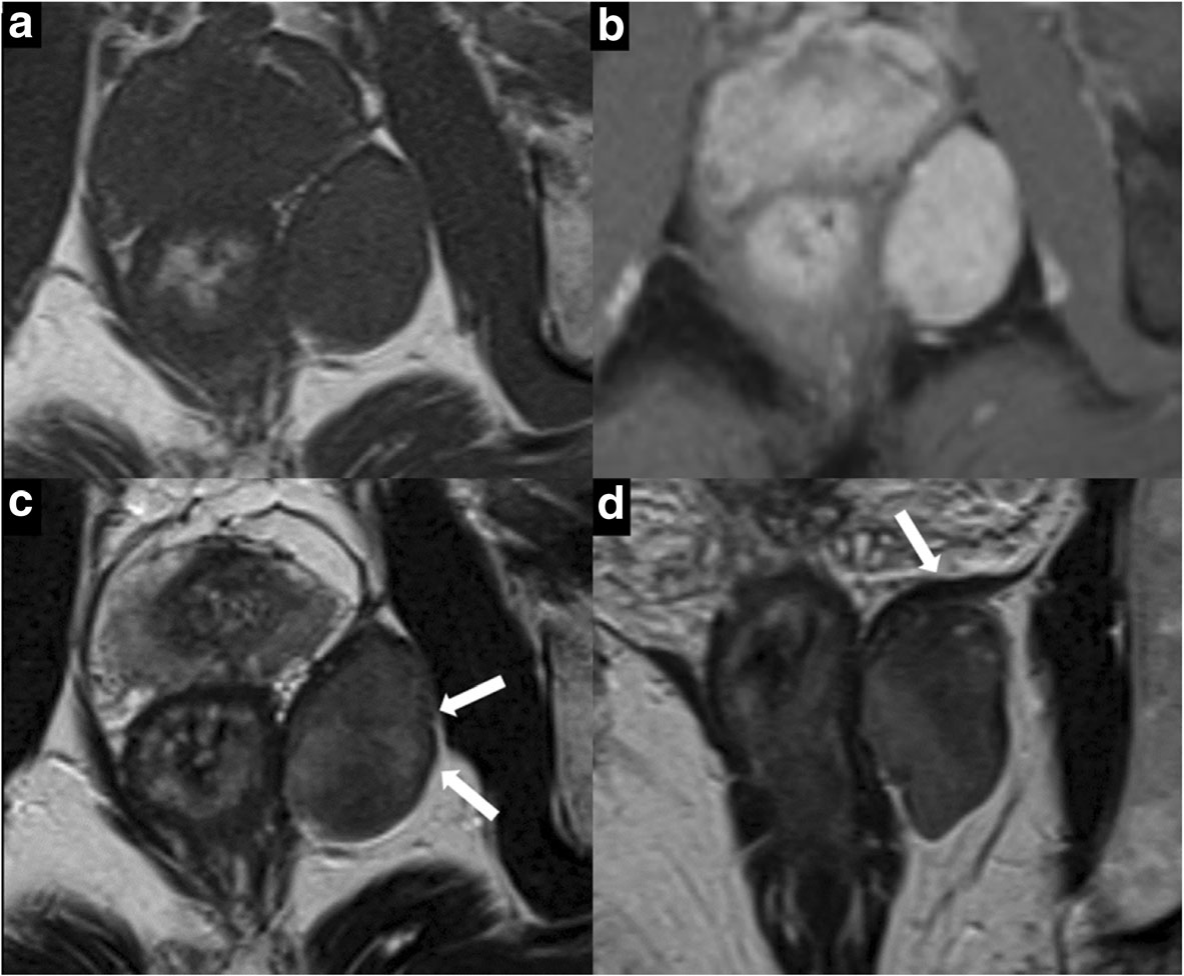
MRI findings. **a** Axial T1-weighted image shows homogenous intermediate signal intensity. **b** Gadolinium contrast-enhanced fat-suppressed T1-weighted image (4 min after contrast material injection) shows homogenous enhancement. **c** Axial T2-weighted image shows heterogeneous intermediate and low signal mixed intensity. The mass contains areas of low signal bands and heterogeneous high signal intensity (white arrows). **d** Coronal T2-weighted image: the tumor is present under the levator ani muscle (white arrow)

Magnetic resonance imaging (MRI) revealed several useful findings for differential diagnosis (Fig. 2). T1-weighted images of the mass showed homogenous intermediate signal intensity (Fig. 2a). T2-weighted images yielded heterogeneous intermediate and low signal intensity (Fig. 2c, d). Images of the mass contained areas of low signal bands (Fig. 2c) and heterogeneous high signal intensity (Fig. 2c, d). The gadolinium contrast-enhanced fat-suppressed T1-weighted images showed homogenous enhancement in the delayed phase (Fig. 2b). Persistent delayed enhancement and low signal bands on T2-weighted images suggested a fibrous component of the mass. High signal intensity on T2-weighted images was suggestive of various components such as myxoid or necrotic cysts.

Although contrast-enhanced CT showed nonspecific findings that were not inconsistent with a benign tumor, an SFT was suspected particularly from the MRI findings. Our patient did not hope for surgery if the tumor was benign; however, most SFTs are benign but have malignant potential; therefore, an ultrasound-guided, transperineal core needle biopsy was performed for a more precise diagnosis to decide on a treatment strategy.

**Fig. 3:**
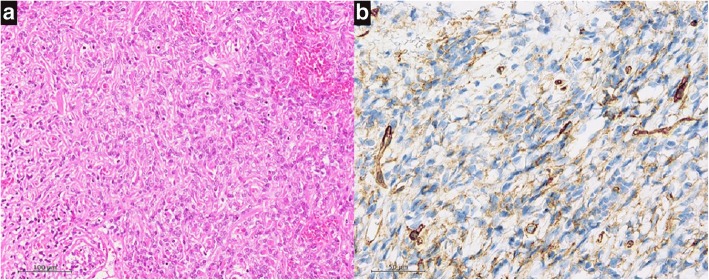
**a** Hematoxylin and eosin staining. Low power magnification (× 200) demonstrates a “patternless” architecture with spindle-shaped cells, abundant stromal collagen bundles, and ectatic vessels. **b** CD34 staining demonstrates diffuse positive staining at high power magnification (× 400)

Microscopic examination showed tumor cells appearing as spindle and fibroblast-like cells within a collagenous stroma (Fig. 3a). Immunohistochemistry identified cluster of differentiation (CD) 34 and vimentin, supporting the diagnosis of an SFT (Fig. 3b).

**Fig. 4:**
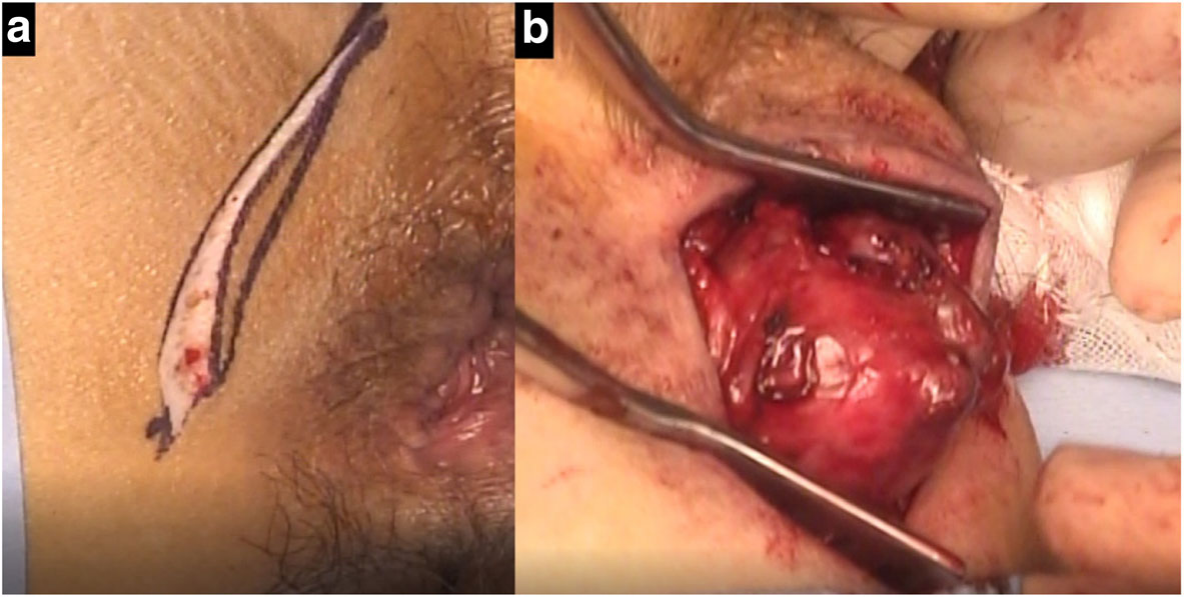
**a** The patient was placed in a prone jackknife position. A vertical incision was made. **b** An intraoperative view demonstrates a capsulized mass

The patient consented to excision of the mass. He was placed in a prone jackknife position, and the tumor was removed transperineally using a posterior approach (modified Kraske procedure). A 5-cm vertical incision was used to approach the mass (Fig. 4a). Following a short split of the gluteus maximus muscle at the cranial part of the incision, a careful blunt dissection was performed while compressing the tumor to the anal side from inside the rectum with the index finger (Fig. 4b). The tumor was separated from the rectal wall, the levator ani muscle, and the external sphincter muscles and avoided cutting into the mass itself. The levator ani muscle, the external sphincter muscles, and rectum were not involved and easily separated from the tumor. Considering the potential for tumor seeding along the needle tract, en bloc excision of the needle tract with the tumor was successfully performed with no complication, and the integrity of the peripheral structures was preserved. The posterior approach provided good exposure without excision of the coccyx. The patient was discharged 5 days after the surgery without complications.

**Fig. 5:**
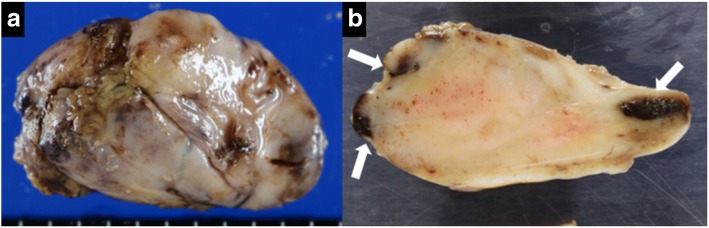
**a** Gross examination of the tumor. **b** Sectioned gross specimen demonstrates a solid, yellowish-white cut surface with hemorrhagic (white arrows) areas

Gross examination of the tumor demonstrated a 53 × 35 × 25-mm, well-defined, completely encapsulated mass (Fig. 5a). The sectioned gross specimen demonstrated a solid, yellowish-white cut surface with hemorrhagic areas (Fig. 5b). Microscopically, the tumor cells appeared as spindle and fibroblast-like cells within a collagenous stroma, and it was consistent with an SFT. Necrosis and cytological atypia were not present. Mitotic activity was one mitosis per ten high power fields (HPF). Immunohistochemical analysis of the tumor cells showed positive results for CD34 and vimentin but negative for CD31, epithelial membrane antigen (EMA), α-smooth muscle antibody (SMA), desmin, and S-100.

There was no local recurrence of the tumor during the year following surgery.

An SFT is a rare neoplasm that occurs in approximately 2.8 of every 100,000 people. Recent advances in electron microscopy and immunohistochemistry have demonstrated a mesenchymal rather than a mesothelial origin of SFTs. While the majority of SFTs occur within the pleura, approximately 30% of SFTs arise in the peritoneal cavity, retroperitoneal soft tissue, and pelvis [1, 2]. However, SFTs occurring in the ischiorectal fossa are extremely rare.

Pelvic SFTs usually occur during the fifth decade of life (age range, 20–70 years) and do not exhibit any gender predilection [3]. Most patients are asymptomatic, but large tumors may result in the compression of adjacent structures. Refractory hypoglycemia (Doege-Potter syndrome) has been reported in 5% of patients [1].

Tumors involving the ischiorectal fossa are most often secondary to direct extensions of primary anorectal, prostate, and sacral tumors [4]. Primary tumors are rare and diverse, benign (e.g., aggressive angiomyxomas [4–6], plexiform neurofibromas [4, 6], GIST [5, 6], leiomyomas [7]) to malignant (e.g., metastatic disease [4], lymphomas [5], liposarcomas [5], epithelioid sarcomas [5]). In these primary tumors, solid well-circumscribed and slow-growing tumors are often benign and tend to be followed up depending on their size. Most SFTs are also benign, but approximately 10–15% of SFTs are malignant, recurring locally or as metastases [5].

Typical SFTs show an architecture characterized by a combination of alternating hypocellular and hypercellular areas separated by thick bands of hyalinized, somewhat keloidal collagen and branching hemangiopericytoma-like vessels. Myxoid changes, areas of fibrosis, and interstitial mast cells are commonly observed. Some SFTs may contain mature adipocytes and/or giant multinucleated stromal cells, thus overlapping morphologically with the so-called lipomatous hemangiopericytomas and giant cell angiofibromas. Malignant SFTs are usually hypercellular lesions showing at least focally moderate to marked cytological atypia, tumor necrosis, numerous mitoses (≥ 4 mitoses per ten HPF) and/or infiltrative margins [8]. Tumor cells in SFTs are characteristically immunoreactive for CD34 (90 to 95% of cases) and CD99 (70%). Twenty to 35% of SFTs are positive for EMA, BCL2, and SMA [8]. Because of their rarity, reports detailing radiological findings of SFTs of the ischiorectal fossa are few [3, 5, 9]. CT scans usually show a smooth-margined, soft-tissue mass with uniform soft-tissue attenuation and heterogeneous enhancement of secondary to myxoid degeneration, hemorrhage, or necrosis [5]. Calcification arising within an SFT is rare and is usually seen only in large tumors [3].

MRI shows intermediate signal intensity on T1-weighted images and heterogeneous high and low signals of mixed intensity with multiple flow voids representing prominent vascular channels on T2-weighted images [3, 5]. On T2-weighted images, areas of low signal intensity suggest mature fibrous tissue containing few cells and abundant collagen stroma. In contrast, areas of high signal intensity suggest edema, cellularity, myxoid degeneration, hemorrhage, or necrosis on T2-weighted images [5]. Intense heterogeneous enhancement that persists in the delayed phase images may be obtained following contrast material administration. Intense arterial enhancement indicates the hypervascular nature of the mass [3]. Large collateral feeding vessels are present in 35% of SFTs; however, the presence of the collateral vessels is not specific, and other hypervascular tumors also show these findings [9]. Persistent delayed enhancement suggests the fibrous nature of the tumor [3]. Hemorrhage [1], tumor necrosis, and infiltrative margins (in approximately 10% of cases) [8] are mostly observed in locally aggressive or malignant tumors.

The differential diagnosis of primary tumors in the ischiorectal fossa with hypervascular and fibrous nature are GIST, leiomyoma, and soft tissue sarcoma. MRI findings based on tissue characterization of tumors can narrow the differential diagnosis; however, they often overlap. Therefore, further accurate and less invasive investigations for diagnosis are necessary to determine an appropriate treatment strategy.

Given the perceived potential for tumor seeding along the needle tract even for SFTs [10, 11], percutaneous biopsy of a potentially malignant lesion is controversial. According to Berger-Richardson’s [12] report that examined seeding after percutaneous needle biopsy for sarcoma, in the setting of a retroperitoneal sarcoma, a review of pooled data from four large tertiary care referral centers demonstrated a 0.37% risk of needle tract seeding. They concluded that the benefits of pretreatment biopsy in patients with mesenchymal tumors outweigh the potential risks of needle tract seeding. They also concluded that although en bloc excision of the needle tract with the primary tumor is often performed, this practice is not associated with improved oncologic outcomes. Buchs et al. [13] described that the biopsy can alter the management of the patient. Certain cases of aggressive angiomyxoma can be treated initially with goserelin acetate (GnRH agonist, Zoladex), some GIST with imatinib, and some desmoids fibromatosis with tamoxifen and sulindac; a reduction in the size of the lesion or a reduction of the local invasion has been observed.

Complete surgical excision is the only established curative treatment for an SFT. However, surgery may become difficult due to extensive adhesions to adjacent vital structures. If complete surgical excision is not possible with an acceptable safety margin, local recurrence is a possibility. Generally, the posterior approaches in the prone jackknife position (modified Kraske procedure) are commonly used for incision of the ischiorectal fossa and presacral tumors [11, 13–16]. The details of approach are dictated by the location and the size of the mass and its relationship with adjacent structures. In the posterior approach, an incision starting from the lower mid-sacrum or the tip of the coccyx to the side of the anus, parasacral incision, or vertebral para-anal incision is used [11, 15–17]. The ipsilateral gluteus maximus and sacrotuberous ligament are divided over the ischial tuberosity and edge of the sacrum, and if not given sufficient exposure for the removal of the tumor, the division of the lower sacrum below the border of the S3 vertebra may follow [11, 15]. This approach can allow entry into the presacral space, at the point proximally above the location of the tumor. Low-lying tumors (the upper limit of the lesion is below the level of S4) can be removed by the posterior approach [11, 16, 17]. For small lesions (less than 5 cm), the lower sacrum can be spared [11, 15, 16]. If the superior border of the tumor can be palpated during the digital examination, the posterior approach should be successful [17]. The posterior approach provides good access to the caudal component, but the major drawbacks are the absence of control over pelvic vessels and the potential for injury to the lateral pelvic nerves [16]. An anterior transabdominal approach is recommended for large and high-lying tumors which extend superiorly through the levator muscles) [13]. In our case, bleeding during surgery was quite limited, but preoperative embolization of highly vascular SFTs may be used to reduce intraoperative blood loss [18, 19]. In unresectable cases, surgical cytoreduction with chemotherapy and radiation therapy might be useful [20, 21]. There is no consensus concerning follow-up for SFTs, but continued long-term surveillance is advised due to the indolent nature of the tumor and the possibility of late recurrence up to 20 years after initial treatment [1, 2].

## Conclusions

Imaging findings reflect the tissue characterization such as hypervascularity and fibrous nature of SFTs and narrow the differential diagnosis. We have presented a rare case of an SFT in the ischiorectal fossa with useful imaging findings for diagnosis, treatment strategy, and successful removal using a posterior approach.

## Abbreviations

CD: Cluster of differentiation
CT: Computed tomography
EMA: Epithelial membrane antigen
GIST: Gastrointestinal stromal tumor
MRI: Magnetic resonance imaging
SFT: Solitary fibrous tumor
SMA: Smooth muscle antibody
